# Asian consensus on diagnostic algorithm for invasive pulmonary fungal diseases in high-burden settings

**DOI:** 10.1515/jtim-2026-0065

**Published:** 2026-08-26

**Authors:** Wenjie Fang, Xinyao Liu, Hazrat Bilal, Mohammad Daud Habib, Ahmed Nasir Hanifi, Nek Wali Shah Momin, Cornelia Lass-Flörl, Chin Samnang, Li An, Cunwei Cao, Min Chen, Zhongju Chen, Shuwen Deng, Li Fan, Jie Gong, Bing Gu, Jian Guo, Xiaoping Huang, Yingqian Kang, Guanzhao Liang, Likai Lin, Jiayun Liu, Weida Liu, Shiyuan Liu, Wei Liu, Zhengtu Li, Zhuozhe Li, Binghuai Lu, Zhiming Lu, Yuxing Ni, Xiaosong Qin, Haibo Qiu, Yiqing Qu, Dongmei Shi, Yi Shi, Xin Su, Hao Tang, Minggui Wang, Yong Wang, Junxue Wang, Wenjuan Wu, Bin Xu, Jinfu Xu, Yingchun Xu, Feng Ye, Yunsong Yu, Liping Zhu, Lin Zhou, Zhiyong Zong, Abdella Gemechu, Surajit De Mandal, Pratibha Gaur, Francesco Barchiesi, Giuseppina Caggiano, Bahareh Abbasi, Hamid Reza Jamaati, Shahla Mohammad Ganji, Sarah A. Ahmed, Khaled Alobaid, Leslie Thian Lung Than, Abdullah M. S. Al-Hatmi, Afreenish Amir, Irum Mehmood, Matiur Rahman, Montarop Sudhadham, Macit İlkit, Pham Quynh Hoa, He Wang, Pham Hong Nhung, Thi Duyen Thi, Wanqing Liao, Xiaojun Huang, Weihua Pan

**Affiliations:** Shanghai Key Laboratory of Molecular Medical Mycology, Center for Basic Research and Innovation of Medicine and Pharmacy, Ministry of Education of the PRC, Shanghai Changzheng Hospital, Naval Medical University, Shanghai, China; Laboratory of Pathogen Research, West China Hospital, Sichuan University, Chengdu, Sichuan Province, China; Jiangxi Key Laboratory of Oncology, Jiangxi Cancer Hospital & Institute, The Second Affiliated Hospital of Nanchang Medical College, Nanchang, Jiangxi Province, China; Pulmonology department, Amiri Medical Complex, Kabul, Afghanistan; Central Public Health Laboratory (CPHL), Ministry of Public Health (MoPH), Kabul, Afghanistan; French Medical Institute for Mothers and Children (FMIC), Kabul, Afghanistan; Institute of Hygiene and Medical Microbiology, ECMM Excellence Center of Medical Mycology, Medical University of Innsbruck, Innsbruck, Austria; Advisors to the Royal Government of Cambodia, Phnom Penh, Cambodia; Department of Respiratory and Critical Care Medicine, Beijing Institute of Respiratory Medicine and Beijing Chao-Yang Hospital, Capital Medical University, Beijing Key Laboratory of Multimodal Intelligent Diagnosis and Treatment System for Respiratory Diseases, Beijing, China; Department of Dermatology and Venereology, The First Affiliated Hospital of Guangxi Medical University, Nanning, Jiangsu Province, China; Department of Dermatology, Shanghai University of Medicine & Health Sciences Affiliated Zhoupu Hospital, Shanghai, China; Department of Microbiology Laboratory, Tongji Hospital, Tongji Medical College, Huazhong University of Science and Technology, Wuhan, Hubei Province, China; Department of Medical Microbiology, The People's Hospital of Suzhou New District, Suzhou, Jiangsu Province, China; Department of Radiology, Changzheng Hospital, Naval Medical University, Shanghai, China; National Key Laboratory of Intelligent Tracking and Forecasting for Infectious Diseases, National Institute for Communicable Disease Control and Prevention, Chinese Center for Disease Control and Prevention, Beijing, China; Laboratory Medicine, Guangdong Provincial People's Hospital (Guangdong Academy of Medical Sciences), Southern Medical University, Guangzhou, Guangdong Province, China; Department of Laboratory Medicine, Shanghai East Hospital affiliated to Tongji University, Shanghai, China; Department of Infectious Diseases, The First Affiliated Hospital of Soochow University, Suzhou, Jiangsu Province, China; Guizhou Key Laboratory of Microbiome and Infectious Disease Prevention & Control, School of Basic Medical Science, Institution of One Health Research, Guizhou Medical University, Gui'an New District, Guizhou Province, China; Department of Medical Mycology, Hospital for Skin Diseases, Institute of Dermatology, Chinese Academy of Medical Sciences and Peking Union Medical College, Nanjing, China; Hospital Management Institute of Wuhan University, Zhongnan Hospital of Wuhan University, Wuhan, Hubei Province, China; Department of Clinical Laboratory Medicine, Xijing Hospital, Air Force Medical University, Xi'an, Shaanxi Province, China; Department of Dermatology and Venereology, Peking University First Hospital; National Clinical Research Center for Skin and Sexually Transmitted Diseases; Beijing Key Laboratory of Innovative Clinical Research and Translation in Skin Diseases; Research Center for Medical Mycology, Peking University, Beijing, China; State Key Laboratory of Respiratory Disease, National Clinical Research Center for Respiratory Disease, Guangzhou Institute of Respiratory Health, The First Affiliated Hospital of Guangzhou Medical University, National Center for Respiratory Medicine, Guangzhou, Guangdong Province, China; Department of pulmonary medicine, Zhongshan hospital. Fudan University, Shanghai, China; Laboratory of Clinical Microbiology and Infectious Diseases, Department of Pulmonary and Critical Care Medicine, Beijing Key Laboratory of Surveillance, Early Warning and Pathogen Research on Emerging Infectious Diseases, National Center for Respiratory Medicine, China-Japan Friendship Hospital, Beijing, China; Department of Clinical Laboratory, Shandong Provincial Hospital Affiliated to Shandong First Medical University, Jinan, Shandong Province, China; Department of Laboratory Medicine, Ruijin Hospital, Shanghai Jiao Tong University School of Medicine, Shanghai, China; Department of Laboratory Medicine, Liaoning Clinical Research Center for Laboratory Medicine, Shengjing Hospital of China Medical University, Shenyang, Liaoning Province, China; Jiangsu Provincial Key Laboratory of Critical Care Medicine, Department of Critical Care Medicine, Zhongda Hospital, School of Medicine, Southeast University, Nanjing, Jiangsu Province, China; Department of Pulmonary and Critical Care Medicine, Qilu Hospital of Shandong University, Jinan, Shandong Province, China; The Laboratory of Medical Mycology & Department of Dermatology, Jining No. 1 People's Hospital, Jining, Shandong Province, China; Department of Respiratory and Critical Care Medicine, Jinling Hospital, Medical School of Nanjing University, Nanjing, Jiangsu Province, China; Department of Respiratory and Critical Care Medicine, Nanjing Drum Tower Hospital, The Affiliated Hospital of Nanjing University Medical School, Nanjing, Jiangsu Province, China; Department of Respiratory and Critical Care Medicine, Shanghai Changzheng Hospital, Naval Medical University, Shanghai, China; Institute of Antibiotics, Huashan Hospital Fudan University, Shanghai, China; Department of Infectious Diseases, Shanghai Changzheng Hospital, Naval Medical University, Shanghai, China; Department of Respiratory and Critical Care Medicine, Tongji Hospital, School of Medicine, Tongji University, Shanghai, China; Department of Clinical Laboratory, State Key Laboratory of Complex Severe and Rare Diseases, Peking Union Medical College Hospital, Chinese Academy of Medical Sciences and Peking Union Medical College, Beijing, China; State Key Laboratory of Respiratory Disease, National Clinical Research Center for Respiratory Disease, Guangzhou Institute of Respiratory Health, The First Affiliated Hospital of Guangzhou Medical University, National Center for Respiratory Medicine, Guangzhou, Guangdong Province, China; Department of Infectious Diseases, Center of General Practice Medicine, Zhejiang Provincial People's Hospital, Affiliated People's Hospital, Hangzhou Medical College, Hangzhou, Zhejiang Province, China; Department of Infectious Diseases, Shanghai Key Laboratory of Infectious Diseases and Biosafety Emergency Response, National Medical Center for Infectious Diseases, Huashan Hospital, Fudan University, Shanghai, China; Department of Laboratory Medicine, Shanghai Changzheng Hospital, Naval Medical University, Shanghai, China; Center of Infectious Diseases, West China Hospital, Sichuan University, Chengdu, Sichuan Province, China; Haramaya University, College of Health and Medical Sciences, School of Medical Laboratory Sciences, Harar, Ethiopia; Department of Microbiology, Shri Davara University, Chhattisgarh, India; Department of Biotechnology & Microbiology, Sri Ramaswamy Memorial University, Delhi-National Capital Region, Sonepat, Haryana, India; Department of Biomedical Sciences and Public Health, Università Politecnica delle Marche, Ancona, Italy; Hygiene Section, Interdisciplinary Department of Medicine University of Bari Aldo Moro Bari, Bari, Italy; Genetic Lab, National Institute for Genetic Engineering and Biotechnology (NIGEB), Tehran, Iran; Mashi Daneshvari Hospital, Chronic Respiratory Diseases Research Center (CRDRC), National Research Institute of Tuberculosis and Lung Diseases (NRITLD), Shahid Beheshti University of Medical Sciences, Tehran, Iran; Department of Molecular Medicine, Medical Biotechnology Institute, National Institute of Genetic Engineering and Biotechnology (NIGEB), Tehran, Iran; Department of Microbiology, Faculty of Medicine, Kuwait University, Kuwait City, Kuwait; Microbiology Department, Mycology Reference Laboratory, Mubarak Al-Kabeer Hospital, Kuwait City, Kuwait; Department of Medical Microbiology, Faculty of Medicine and Health Sciences, University Putra Malaysia, Selangor, Malaysia; Natural and Medical Sciences Research Center, University of Nizwa, Nizwa, Oman; Rawalpindi Medical University, Rawalpindi, Pakistan; Hayatabad Medical Complex Peshawar, Peshawar, Pakistan; Health Services Academy, Islamabad, Pakistan; Department of Biology, Faculty of Science and Technology, Suan Sunandha Rajabhat University, Bangkok, Thailand; Division of Mycology, Department of Microbiology, Faculty of Medicine, Çukurova University, Adana, Türkiye; Mycology and Parasitology Department, National Hospital of Dermatology and Venereology, Hanoi, Vietnam; Dynamiker Sub-Center of Beijing Key Laboratory for Mechanisms Research and Precision Diagnosis of Invasive Fungal Disease, Tianjin, China; Department of Microbiology Hanoi Medical University, Hanoi, Vietnam; Department of Dermatology, 108 Military Central Hospital, Hanoi, Vietnam; Peking University People's Hospital, Peking University Institute of Hematology, Beijing, China

**Keywords:** invasive pulmonary fungal diseases, diagnostic algorithm, consensus, immunocompromised host

## Abstract

Invasive pulmonary fungal diseases (IPFD) continue to posing an increasing clinical and public health burden on immunocompromised populations. In many Asian settings, the diagnosis of IPFD faces substantial challenges driven by the high prevalence of comorbidities, including poorly controlled diabetes, tuberculosis (TB), and human immunodeficiency virus (HIV) infection, considerable heterogeneity in local pathogens, and disparities in access to diagnostic resources. To address these unmet needs, experts from 18 countries collaboratively developed this consensus, which provides a diagnostic algorithm tailored to high-burden Asian settings based on existing international and regional guidelines. When clinical manifestations are atypical or diagnostic clues are limited, the algorithm prioritizes evaluation for common and regionally prevalent IPFD, followed by stepwise expansion to other potential fungal pathogens. In addition, this consensus outlines the regional accessibility of different diagnostic modalities across Asia. This consensus focuses exclusively on optimization of the diagnostic algorithm and does not provide specific recommendations regarding antifungal therapy. Notably, improved diagnosis of IPFD through this algorithm may contribute to better patient outcomes and strengthened public health strategies in high-burden regions.

## Introduction

Invasive pulmonary fungal diseases (IPFD) refer to a group of diseases in which fungi invade the pulmonary parenchyma or pulmonary vasculature, resulting in deep-seated infection. These diseases include invasive pulmonary aspergillosis (IPA), pulmonary cryptococcosis, and Pneumocystis jirovecii pneumonia (PJP), among others. Xia *et al*.^[1]^ reported that pulmonary fungal infections account for approximately 5.62 million new cases and more than 45,000 deaths annually, with regions of lower sociodemographic index continuing to shoulder a disproportionately high disease burden.

In 2022, the World Health Organization (WHO) released its first Fungal Priority Pathogen List, categorizing common IPFD pathogens, such as *Aspergillus fumigatus* and *Cryptococcus neoformans*, as members of the “Critical Priority Group” and calling for enhanced surveillance and improved diagnostic capacity.^[2]^ However, implementation of this global initiative in many Asian high-burden settings remains challenging for several reasons. First, Asia possesses unique host characteristics. For example, poor glycemic control among diabetic patients in South Asia contributes to a high regional incidence of mucormycosis. Second, the distribution of fungal pathogens in Asia is distinct, with pathogens such as *Talaromyces marneffei* being endemic in Southeast Asia.^[3]^ Third, IPFD is frequently misdiagnosed as tuberculosis (TB), malignancy, or bacterial pneumonia, with particular concern regarding misdiagnosis as TB in high-burden Asian regions. Fourth, accessibility to diagnostic technologies remains uneven, and disease surveillance systems are often inadequate.

Collectively, these factors highlight a persistent gap between existing guidelines and their practical applicability in high-burden Asian settings. To address this gap, this consensus was jointly developed by 76 experts from 18 countries to guide clinical practice in these settings. In contrast, several high-income Asian countries, including Japan, South Korea, and Singapore, generally adhere to established guidelines.^[4]^ These countries not only possess well-developed diagnostic and treatment systems but are also not considered core high-burden IPFD regions in Asia. Given the high mortality associated with delayed diagnosis and the marked diversity of pathogens and host characteristics across high-burden Asian regions, this consensus proposes a diagnostic algorithm tailored to regional epidemiological characteristics to facilitate timely identification of IPFD while remaining aligned with existing guidelines and clarifying diagnostic sequencing and testing strategies.

## Methods

The World Society of Chinese Medical Mycologists initiated this consensus and subsequently engaged experts from multiple disciplines, including infectious diseases, critical care medicine, respiratory medicine, laboratory medicine, mycology, and health economics. This consensus is registered with the Guidelines International Network and complies with the standards of the Reporting Items for Practice Guidelines in Healthcare (RIGHT) checklist.

“Asian high-burden regions” refers to areas or healthcare environments characterized by a substantial IPFD burden and limited or uneven diagnostic resources. Given the considerable variation in IPFD burden and diagnostic capacity both between and within countries, the algorithm should be adapted according to regional epidemiology and local resources. The core characteristics of high-burden settings are as follows: (1) the incidence and mortality of IPFD are significantly higher than the global average, or specific subtypes exhibit local hyperendemicity even when overall disease levels do not reach this threshold; (2) medical resources are unevenly distributed, advanced diagnostic technologies have limited accessibility, and standardization of clinical diagnosis and treatment is insufficient; and (3) although high-income Asian countries with well-established healthcare systems generally exhibit a lower overall IPFD burden, local endemicity or regional outbreaks may still occur.

The literature regarding IPFD diagnosis was searched in the Cochrane Library, Embase, and PubMed using a predefined search strategy provided in Supplementary Material 1. To ensure the relevance of evidence to the target population, the literature search prioritized studies conducted in the above-defined Asian high-burden regions, focusing on epidemiological characteristics, accessibility of diagnostic resources, and the local distribution of IPFD subtypes.

The search included studies published up to August 2025.

The consensus development process consisted of two rounds of face-to-face and online discussions, followed by email-based review. Consensus was considered achieved when at least 75% of participating experts agreed. This consensus will be updated every five years.

## Pathogens

In certain Asian countries, the incidence of specific IPFD differs substantially from that observed in other regions of the world because of unique pathogen prevalence, underlying diseases, environmental conditions, and medical practices. Therefore, based on Asia-specific epidemiological evidence, this consensus classifies infections caused by these pathogens into “high-probability IPFD” and “low-probability IPFD” categories.

Clinicians may prioritize the diagnostic workup for different IPFD according to the stratified pathogen recommendations presented in Table 1, together with the classification criteria specified in Table 2. For example, mucormycosis is generally regarded as a low-probability IPFD in most global settings; however, the estimated prevalence of mucormycosis in India is approximately 70-fold higher than the global average because of the high prevalence of diabetes and widespread glucocorticoid use.^[5]^ Based on local epidemiological patterns, populations in such regions carry an elevated risk of mucormycosis, which should therefore be reclassified as a high-probability IPFD in these settings.^[6,7]^ Furthermore, during fungal infection outbreaks, pathogens exhibiting a marked increase in incidence or clustered case presentations should also be reclassified from low-probability to high-probability IPFD. Such pathogens include *Candida auris*, *Candida parapsilosis*, *Mucorales*, *Sporothrix*, *Blastomyces*, *Histoplasma*, and *Coccidioides*.^[8,9]^

**Table 1:** Recommended stratified pathogen list for high- and low-probability IPFD

IPFD		Pathogens	Epidemiology	References
High-probability IPFD	Invasive pulmonary aspergillosis	Predominantly *Aspergillus fumigatus*, followed by *A. flavus*, *A. niger*, *A. terreus*	Globally, *A. fumigatus* is the predominant species. In South Asia, parts of the Middle East, and Southeast Asia, *A. flavus* is more prevalent.	[39]
	Pulmonary cryptococcosis	Mainly *Cryptococcus neoformans* species complex; *C. gattii* species complex is less common	*C. neoformans* is epidemiologically associated with pigeon droppings. In China, the majority of cases occur in non-HIV-infected patients, with the ST5 genotype being the most prevalent. *C. gattii* is primarily distributed in tropical/subtropical regions.	
	Pneumocystis pneumonia	*Pneumocystis jirovecii*	Prevalent among HIV/AIDS patients and individuals receiving immunosuppressive therapy.	
Low-probability IPFD	Pulmonary mucormycosis*	Predominantly *Rhizopus*, *Mucor*, and *Lichtheimia* species; less common: *Rhizomucor*, *Cunninghamella*, *Syncephalastrum*, and *Apophysomyces* species	Globally, *Rhizopus arrhizus* is most prevalent species. In South Asia: *R. arrhizus* (most prevalent), *A. variabilis* (second most common), with increasing incidence of *R. microsporus* and *R. homothallicus*.	[5,21]
	Endemic mycoses	Talaromyces marneffei*, *Paracoccidioides*, *Blastomyces*, *Coccidioides*, *Emergomyces*, *Histoplasma*, and *Sporothrix* species	*T. marneffei*: endemic to tropical and subtropical Asia. *Histoplasma*: reported cases in Southeast Asia and southern China. *Emergomyces*: *Es. orientalis* is the predominant species in Asia, with occasional reports of *Es. pasteurianus*; *Blastomyces*: predominantly distributed in North America and Africa, with sporadic cases in Asia. *Sporothrix*: Thailand/Vietnam, mainly lymphocutaneous linked to farming/animal contact	[11,35,36,38,40]
	Candida pneumonia	*C. albicans*, *C. parapsilosis*, *C. glabrata*, *C. tropicalis*, *C. krusei* (syn. *Pichia kudriavzevii*), *C. auris* (syn. *Candidozyma auris*) and others	–	–
	Other rare mycoses	*Fusarium*, *Alternaria*, *Scopulariopsis brevicaulis*, *Gliocladium*, *Penicillium*, *Paecilomyces*, *Talaromyces* species excluding *T. marneffei*	–	–

*In specific regions, these infections may be classified as high-probability IPFD. IPFD: invasive pulmonary fungal disease.

**Table 2:** Classification criteria for high- and low-probability IPFD

Category	Diagnostic criteria
High-probability IPFD	A compatible clinical syndrome is present, with at least one major trigger met from each of the three following domains: • Host predisposition factors (*e.g*., neutropenia, hematologic malignancy, solid organ transplantation, prolonged corticosteroid or other potent immunosuppressive therapy, advanced HIV disease); • Epidemiological or exposure context (*e.g*., recent travel or residence in an endemic area, environmental exposure, healthcare-associated outbreak, relevant animal/soil exposure); • Syndrome-specific clinical-radiological features (*e.g*., nodules with halo sign or cavitation for invasive pulmonary aspergillosis; diffuse ground-glass opacities for PJP; meningoencephalitic syndrome for cryptococcosis; rapidly progressive necrotizing rhino-orbito-cerebral disease for mucormycosis).
Low-probability IPFD	No compatible clinical symptoms, minimal host risk, or radiological and epidemiological findings do not support an invasive fungal aetiology.

IPFD: invasive pulmonary fungal disease; PJP: Pneumocystis jirovecii pneumonia.

## Host-Related factors

The development of IPFD is closely associated with the host immune status and underlying comorbidities. Common high-risk factors include neutropenia, high-dose or prolonged glucocorticoid therapy and other immunosuppressive treatments, hematopoietic stem cell transplantation or solid organ transplantation, malignancies, congenital immunodeficiencies, acquired immunodeficiency syndrome (AIDS), end-stage organ failure, exposure to specific medications, chronic lung diseases, decompensated liver cirrhosis, major surgery or intestinal barrier disruption, and severe viral pneumonia. Table 3 summarizes the host-related factors associated with different subtypes of IPFD.

**Table 3:** Host-related factors for IPFD

Type of IPFD	High-risk population
Invasive pulmonary aspergillosis	Patients with hematologic malignancies, aplastic anemia, hematopoietic stem cell or lung transplant recipients, severe COVID-19 or influenza, chronic granulomatous disease, long-term immunosuppressive therapy or high-dose glucocorticoid administration, chronic obstructive pulmonary disease, tuberculosis, bronchiectasis, or sarcoidosis
Pulmonary cryptococcosis	HIV-infected patients; individuals with tuberculosis, hepatic or renal disease, diabetes mellitus, or malignancies; patients receiving glucocorticoids or immunosuppressants; and those exposed to soil or avian excreta. Notably, immunocompetent individuals may also develop infection
Pneumocystis pneumonia	HIV-infected individuals, solid organ transplant recipients, patients with leukemia, lymphoma, or autoimmune diseases, those on high-dose glucocorticoids or long-term immunosuppressive regimens, and cancer patients undergoing chemotherapy
Pulmonary mucormycosis	Patients with poorly controlled diabetes mellitus, hematologic malignancies, or recipients of solid organ or hematopoietic stem cell transplantation
Endemic mycoses	Travelers to or residents of regions endemic for specific fungal pathogens (*e.g*., *Talaromyces marneffei* in Guangxi/Guangdong Provinces, *Histoplasma* species in the Yangtze River Basin), HIV-infected individuals, and other immunocompromised populations
Pulmonary candidiasis	ICU patients, those with indwelling central venous catheters, recipients of total parenteral nutrition, patients with acute kidney injury, a history of septic shock, or exposure to aminoglycoside antibiotics
Other rare fungal diseases	Patients with hematologic malignancies, ICU patients, those on long-term antifungal therapy, individuals with indwelling central venous catheters, and patients with underlying genetic susceptibility

IPFD: invasive pulmonary fungal disease; ICU: intensive care unit.

Population health profiles and disease patterns vary considerably across Asian countries, resulting in distinct distributions and risk characteristics among populations susceptible to IPFD.

In South Asia, particularly in India, poorly controlled diabetes mellitus is the most prevalent risk factor for mucormycosis, especially in patients with diabetic ketoacidosis, thereby warranting heightened clinical vigilance. However, as observed during the coronavirus disease 2019 (COVID-19) pandemic, although rhino-orbital-cerebral mucormycosis (ROCM) has been widely reported in these regions, pulmonary mucormycosis is likely underrecognized in settings lacking routine diagnostic capacities, such as lower respiratory tract sampling.^[10]^ In patients presenting with fever, hemoptysis, and pulmonary infiltrates, particularly those with poorly controlled diabetes mellitus even in the absence of hematologic disorders, clinicians should maintain a high index of suspicion for mucormycosis at an early stage.^[5, 6, 7]^

In regions with a high prevalence of TB, including India, Indonesia, and China, and in areas where fungal-specific diagnostic assays and high-resolution computed tomography (CT) are not widely accessible, multiple subtypes of IPFD are frequently misdiagnosed as pulmonary TB. These conditions include pulmonary aspergillosis, cryptococcosis, PJP, blastomycosis, histoplasmosis, and talaromycosis.^[11]^ In patients presenting with chronic pulmonary symptoms, cavitary lesions, pulmonary nodules, hemoptysis, or poor responses to antibacterial or anti-TB therapy, TB testing and fungal investigations should be performed in parallel when clinically indicated.

Although the prevalence of human immunodeficiency virus (HIV) infection in Asia is lower than that in sub-Saharan Africa, the large population size in the region sustains a substantial burden of HIV-associated fungal infections. In several Southeast Asian countries, particularly Thailand, Myanmar, and Cambodia, IPFD is highly prevalent among HIV-infected individuals. Patients with CD4 counts below 200/μL are at particularly high risk and frequently present with multiple co-infections, such as PJP combined with TB or cryptococcosis combined with *Talaromyces marneffei* infection. Therefore, parallel testing for multiple pathogens is essential in clinical practice.

## Clinical manifestations

The clinical manifestations of IPFD are often nonspecific. Patients commonly present with fever, cough, hemoptysis, chest pain, and dyspnea. The specific manifestations depend on the host immune status and the causative pathogen. Because bacterial pneumonia, viral pneumonia, and pulmonary TB are more common, the imaging and clinical features of IPFD frequently overlap with those of these infections, thereby contributing to delays in early diagnosis. This section summarizes the key clinical characteristics that influence diagnosis and provides a foundation for the subsequent diagnostic approach.

The clinical presentation of IPA is closely associated with the severity of host immunosuppression. Patients with neutropenia often present with persistent fever, and the disease may progress rapidly because of its angioinvasive nature, with mortality rates exceeding 50% in this population. Early chest CT findings may demonstrate the halo sign. During neutrophil recovery or early antifungal therapy, lesions may enlarge and develop into air crescent signs or cavitary lesions. In non-neutropenic patients, fever may be mild, and imaging findings often reveal reticular nodules or infarct-like consolidation, which are nonspecific. Concurrent infections are common and may further complicate imaging interpretation. Patients with hematologic malignancies, hematopoietic stem cell transplantation, chronic lung disease, or prior TB require careful evaluation for IPA.^[12, 13, 14, 15]^

Pulmonary cryptococcosis is often asymptomatic in immunocompetent individuals and commonly presents as pulmonary nodules. However, in immunocompromised individuals or patients with HIV infection, diffuse pulmonary infiltrates and central nervous system (CNS) involvement may occur. Cryptococcal antigen testing is useful for diagnosis.^[16,17]^

Among HIV-positive patients, PJP typically progresses slowly and presents with symptoms such as dyspnea, nonproductive cough, and diffuse ground-glass opacities on imaging. In contrast, PJP progresses rapidly in HIV-negative patients and often results in severe respiratory failure accompanied by atypical radiological manifestations.^[18,19]^

Pulmonary mucormycosis usually has an acute onset and presents with fever, cough, chest pain, or hemoptysis. In Asia, poorly controlled diabetes mellitus, particularly diabetic ketoacidosis, represents a major risk factor.^[12,20,21]^

In Asia, endemic mycoses exhibit distinct epidemiological patterns. In patients with HIV infection, *Talaromyces marneffei* infection may cause fever, weight loss, hepatosplenomegaly, cutaneous lesions, and occasional pulmonary involvement. Acute histoplasmosis often mimics viral respiratory illness, whereas subacute cases typically present with cough and hilar lymphadenopathy. Severe cases may disseminate systemically. *Emergomyces* infection mainly affects severely immunocompromised patients and may cause skin lesions, pulmonary infiltrates, and multisystem organ involvement.^[11]^

The clinical manifestations of pulmonary candidiasis are nonspecific. The disease may occur as either a primary pulmonary infection or as part of hematogenous dissemination. Most infections occur in patients with systemic immunosuppression or impaired local pulmonary defense mechanisms and typically present with fever, cough, and dyspnea, which are indistinguishable from those of other pulmonary infections. Isolation of *Candida* species from sputum or bronchoalveolar lavage fluid (BALF) cannot reliably distinguish colonization from true infection, and definitive diagnosis requires histopathological confirmation. This condition is prone to both misdiagnosis and overdiagnosis in clinical practice and should be interpreted cautiously in the context of mycological findings and host-related factors.^[22]^ Notably, overdiagnosis frequently results in unnecessary antifungal therapy, thereby increasing the risk of antifungal resistance and imposing additional burdens on healthcare resources.

IPFD caused by rare fungal pathogens is infrequently reported and generally presents with nonspecific clinical and radiological manifestations. Diagnosis typically depends on histopathological examination or molecular testing and should be considered when fungal infection is strongly suspected despite atypical results from routine diagnostic tests.^[23,24]^

## Diagnostic algorithm for IPFD

**Figure 1:**
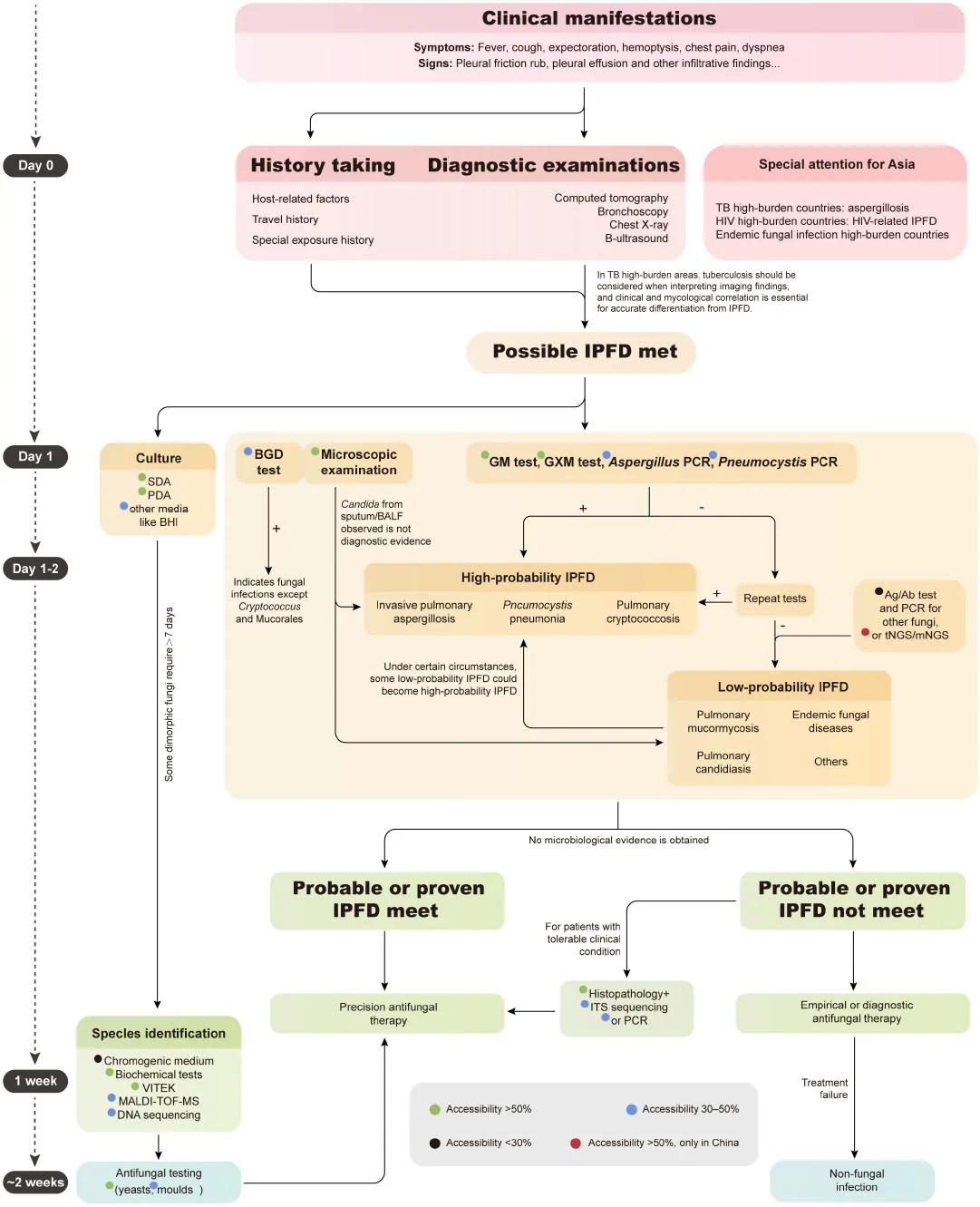
Diagnostics algorithm for Invasive pulmonary fungal diseases. This figure depicts the standardized diagnostic algorithm for IPFD in high-burden Asian countries, presenting a time-aligned workflow (spanning Day 0 to 2 weeks) that integrates clinical evaluation, mycological testing, IPFD probability stratification, and corresponding management strategies—with supplementary region-specific clinical considerations tailored to the Asian context, where “accessibility” refers to the availability proportion of diagnostic methods in these high-burden settings. IPFD: invasive pulmonary fungal diseases; BDG: β-D-glucan; GM: galactomannan; GXM: glucuronoxylomannan; PCR: polymerase chain reaction; NGS: next-generation sequencing; tNGS: targeted next-generation sequencing; TB: tuberculosis; BALF: bronchoalveolar lavage fluid; MALDI-TOF MS: matrix-assisted laser desorption/ionization time-of-flight mass spectrometry.

This consensus proposes a standardized diagnostic algorithm for Asia based on existing guidelines and integrated regional epidemiological characteristics, including at-risk populations, pathogen prevalence, and disparities in healthcare resources (Figure 1). The algorithm is intended to guide diagnostic escalation and reassessment and does not define antifungal drug selection, dosing, or treatment duration. Given the marked variability in diagnostic capacity across high-burden Asian settings, the algorithm should be applied in a resource-adapted manner, with escalation or referral pathways implemented when advanced diagnostic methods are unavailable.

It is important to recognize that no single diagnostic modality demonstrates sufficient sensitivity and specificity for invasive fungal infections in routine clinical practice, and microbiological confirmation is often difficult to obtain. Diagnostic performance is influenced by specimen type, timing of sampling, prior antifungal exposure, and assay-specific limitations, which may result in discordant findings. Accordingly, results should be interpreted probabilistically by integrating pre-test probability, specimen quality, and signal strength, with repeat or confirmatory testing considered in clinically critical or discordant cases.^[25]^

## Identifying patients for mycological testing

The algorithm begins with clinical assessment. Evaluation of underlying diseases, immunosuppressive status, travel and exposure history, and imaging findings, preferably chest CT, helps determine the likelihood of invasive pulmonary fungal disease when clinically suspected. Bronchoscopy may be performed to obtain respiratory specimens when necessary, whereas ultrasound is primarily used to evaluate pleural effusions or assist in sampling peripheral pulmonary lesions. The European Organization for Research and Treatment of Cancer/Mycoses Study Group Education and Research Consortium (EORTC/MSGERC) host and clinical criteria for probable disease should then be assessed. These criteria perform optimally in classically immunocompromised hosts but may have limited applicability in intensive care unit (ICU) or non-neutropenic settings, in which setting-adapted clinical pathways should be considered.

In diagnostically uncertain cases, particularly in patients with persistent fever, nonspecific CT findings, chronic obstructive pulmonary disease (COPD), prior TB, severe viral pneumonia, or corticosteroid exposure, IPFD should not be excluded prematurely. Reassessment, repeat sampling, or BALF-based or molecular testing should be considered when clinical suspicion persists. Imaging may reveal suggestive features. The halo sign is an early radiological feature highly suggestive of IPA, the air crescent sign is a late finding strongly indicative of IPA, and the reverse halo sign is frequently associated with mucormycosis. Although these findings are not specific to individual fungal pathogens, they possess substantial diagnostic value for their respective diseases. By integrating regional epidemiology with individual exposure history, clinicians can identify the most likely pathogens and prioritize mycological diagnostic tests for high-probability pathogens.

## Defining the diagnostic strategy for mycological testing

The diagnostic process should proceed in a stepwise manner according to timing and accessibility (Supplementary Table S1), with priority given to testing methods targeting pathogens associated with high-probability IPFD. Herein, “accessibility” refers to the availability of diagnostic methods in high-burden Asian settings, and detailed information regarding the accessibility of each recommended testing modality is provided elsewhere.^[26]^

Preferred diagnostic methods are those capable of providing rapid results, including microscopic examination, fungal culture, *Aspergillus* galactomannan (GM) testing, *Cryptococcus* glucuronoxylomannan (GXM) antigen testing, and targeted reverse transcription polymerase chain reaction (RT-PCR) assays, such as those for *Aspergillus* and *Pneumocystis*. If initial tests are negative but clinical suspicion remains high, repeat specimen collection and additional investigations, including BALF GM testing, histopathological examination, or molecular sequencing, may be considered.

In some high-resource settings, including parts of China, metagenomic next-generation sequencing (mNGS) and targeted next-generation sequencing (tNGS) are increasingly used; however, their accessibility and cost vary substantially across Asian regions. These assays may serve as supplementary tools for identifying difficult-to-culture or atypical pathogens, although their results should be interpreted cautiously because of contamination, colonization, and the lack of standardized diagnostic thresholds. If repeated testing remains negative, additional investigations for pathogens categorized as “low-probability IPFD” may be considered.

## Integrating diagnostics with clinical decision-making and reporting

Cultures should be obtained early and processed in parallel with other diagnostic tests. Once positive results are obtained, the recovered fungus should be identified promptly at the species level, and findings should be communicated in a time-sensitive manner to facilitate clinical management. Where available, antifungal susceptibility testing should be performed to support result interpretation and local surveillance efforts.

If prior investigations are nondiagnostic and the patient’s clinical condition permits, histopathological examination with or without internal transcribed spacer (ITS) sequencing or polymerase chain reaction (PCR) may be performed to confirm tissue invasion. The algorithm emphasizes the timeliness of diagnosis and clinical decision-making, aiming to enable appropriate therapy to be initiated or adjusted within 1 to 2 days when clinically indicated.

Building upon the standardized diagnostic algorithm described above, tailored mycological testing strategies for suspected cases of individual IPFD entities are outlined below.

## Patients with suspected IPA

Priority should be given to rapid diagnostic methods, including microscopy, culture, GM testing, and *Aspergillus*-specific PCR. While awaiting species identification by culture, if *Aspergillus terreus* is identified, reduced susceptibility to amphotericin B should be considered, and azole susceptibility should be assessed on an isolate-specific basis whenever feasible, with therapy adjusted accordingly.^[27]^ Laboratories are encouraged to perform antifungal susceptibility testing for *Aspergillus* isolates. If the patient's condition permits and additional diagnostic confirmation is required, histopathological examination may be considered; however, it is not recommended as a first-line approach in patients with bleeding risk or poor general condition.^[12, 13, 14, 15,28,29]^

Notes:

1. Routine combined use of *Aspergillus*-specific PCR and GM testing is recommended to improve the detection rate of IPA. Performing at least two PCR assays is advised to enhance diagnostic specificity and yield.
2. In patients with respiratory failure, those already receiving antifungal therapy, patients with high clinical suspicion of IPA but negative serum GM results, or those with potential false-positive interference, BALF GM testing is recommended as the preferred approach, with *Aspergillus*-specific immunoglobulin G (IgG) testing serving as a complementary adjunct.^[30]^
3. In patients already receiving antifungal therapy, the sensitivity of GM testing may be reduced, and mold-active therapy may also decrease PCR yield. Therefore, negative results from either assay should be interpreted cautiously, and BAL-based testing should be prioritized for the diagnosis or exclusion of IPA whenever feasible.
4. For paraffin-embedded tissue sections demonstrating suspected *Aspergillus* elements on microscopic examination, *Aspergillus* PCR testing is recommended.

## Patients with suspected PJP

Rapid diagnostic methods, including microscopy, pathogen-specific PCR, and *β*-D-glucan (BDG) testing, are recommended as first-line approaches. If necessary and feasible, histopathological examination may also be considered.^[18,19,31]^

Notes:

1. Detection of organisms by silver staining using Grocott-Gomori methenamine silver staining in BALF or lung biopsy specimens remains the gold standard for the diagnosis of PJP. However, these methods have limited sensitivity, and negative results cannot exclude the diagnosis.
2. Routine combined use of *Pneumocystis*-specific PCR and BDG testing is recommended. PCR provides high sensitivity and enables species identification but cannot reliably distinguish active infection from colonization, whereas negative BDG results make active PJP less likely but do not exclude disease. Therefore, results should be interpreted in conjunction with host-related risk factors, imaging findings, and microbiological evidence when available.

## Patients with suspected pulmonary cryptococcosis

Rapid diagnostic approaches, including direct microscopy, India ink staining, culture, and cryptococcal GXM antigen testing, are recommended as first-line methods. For patients presenting with CNS symptoms, lumbar puncture should be performed promptly, and cerebrospinal fluid should be analyzed for opening pressure, biochemical parameters, and mycological investigations, including India ink staining, culture, and GXM antigen testing. If necessary and feasible, histopathological examination may also be considered.^[16,17]^

Notes:

1. Routine combined use of India ink staining, fungal culture, and GXM antigen testing is recommended. In addition, BDG testing, GM testing, and multiplex PCR assays for high-risk IPFD pathogens may be considered to exclude other fungal infections.
2. Cultured isolates should undergo molecular identification to differentiate *Cryptococcus gattii* complex from *Cryptococcus neoformans* complex, as these infections differ substantially in epidemiology, clinical manifestations, treatment strategies, mortality, and sequelae.
3. Routine antifungal susceptibility testing is not recommended for patients initiating therapy; however, susceptibility testing is recommended in cases of treatment failure.^[32,33]^

## Patients with suspected pulmonary mucormycosis

Respiratory specimens should be collected as early as possible before initiation of antifungal therapy for microscopy and culture. Concurrent GM testing and multiplex PCR assays targeting high-probability IPFD pathogens are recommended to exclude other common fungal infections. If necessary and feasible, histopathological examination may be performed, and the presence of characteristic fungal structures on microscopic examination may serve as confirmatory evidence. In China, tNGS or mNGS may be used as supplementary diagnostic tools for pulmonary mucormycosis.^[6,7,12,20,21]^

Notes:

1. Direct microscopy is a key diagnostic tool for pulmonary mucormycosis, with particular attention given to hyphal characteristics such as septation, branching angle, and hyphal width.
2. Histopathological examination has high diagnostic value; however, mucoralean hyphae may morphologically resemble *Aspergillus*. Confirmation by experienced personnel is recommended, and further differentiation may be achieved using *Aspergillus*-specific PCR or Sanger sequencing.^[31,34]^
3. Cultured isolates should undergo molecular identification, such as microbial mass spectrometry or Sanger sequencing. Notably, Mucorales infections may yield negative culture results.
4. Routine antifungal susceptibility testing is not recommended at the initiation of therapy.

## Patients with suspected endemic mycoses

Rapid diagnostic methods, including microscopy, culture, and BDG testing, are recommended as first-line approaches. Concurrent GM testing and multiplex PCR assays for high-probability IPFD pathogens may be performed to exclude other common fungal infections. If necessary and feasible, histopathological examination may be conducted, and the observation of characteristic fungal structures on microscopic examination may serve as confirmatory evidence. In China, tNGS or mNGS may be used as supplementary diagnostic tools for endemic mycoses.^[35,36]^

Notes:

1. GM testing is recommended as a nonspecific adjunctive diagnostic tool for *Histoplasma* or *Talaromyces marneffei* infection in patients with HIV infection. Owing to antigen cross-reactivity, GM positivity cannot reliably differentiate *Histoplasma* infection from *Aspergillus* infection; therefore, species-specific PCR or histopathological examination is required for confirmation. GM testing has particular diagnostic value in advanced HIV disease.
2. Compared with mNGS, cost-effective tNGS panels that include endemic fungal pathogens should be prioritized.
3. Clinical benefits must be balanced against laboratory biosafety risks. Although culture from sterile sites may serve as a diagnostic gold standard, the prolonged incubation period required for endemic fungi, typically ≥ 4 weeks (occasionally up to 6 weeks), limits its utility in guiding early therapy. When highly pathogenic fungi such as *Histoplasma*, *Coccidioides*, or *Blastomyces* have already been identified by direct microscopy, histopathological examination, or high-throughput sequencing, discontinuation of further culture or small-scale cultivation is recommended to reduce the risk of laboratory dissemination.

## Patients with suspected pulmonary candidiasis

Priority should be given to microscopy, culture, BDG testing, GM testing, and multiplex PCR for high-probability IPFD pathogens to exclude other common fungal infections. If the patient’s condition allows, histopathological examination may also be considered to confirm the diagnosis.^[22,37]^

Notes:

1. A definitive diagnosis of pulmonary candidiasis requires both the presence of pathogenic *Candida* in lung biopsy tissue and histological evidence of inflammation. Given the rarity and diagnostic difficulty of this disease, the recommended approach is to first evaluate for systemic (extrapulmonary) *Candida* infection and to rule out other pulmonary pathogens. If the diagnosis remains uncertain and the patient’s general condition permits with minimal bleeding risk, lung biopsy may be performed.
2. *Candida* isolated from sputum or BALF alone should not be considered diagnostic of *Candida* pneumonia and should not by itself prompt antifungal therapy, particularly in immunocompetent patients who do not otherwise meet diagnostic criteria, to avoid overtreatment.
3. In immunocompromised patients who do not yet meet full diagnostic criteria but satisfy clinical suspicion, respiratory isolation of *Candida* spp. alone does not establish invasive disease; evaluation should prioritize evidence of invasive candidiasis (*e.g*., candidemia or sterile-site infection) and, where feasible, tissue confirmation.

## Patients with suspected emerging, rare, or mixed pulmonary fungal infections

Priority should be given to microscopy, culture, and BDG testing. Simultaneous GM testing and multiplex PCR for high-probability IPFD pathogens are recommended to exclude more common fungal infections. If the patient’s condition allows, histopathological examination should be performed promptly to confirm the diagnosis. While these mycological tests can provide preliminary evidence of fungal infection, they may not identify the specific pathogen. Therefore, it is recommended to combine repeated conventional testing with mNGS or tNGS for further diagnostic clarification.^[23,24,32,38]^

Notes:

1. It is recommended to perform mNGS on DNA extracted from the patient’s respiratory specimens. If no relevant pathogens are detected, the raw sequencing data should be reviewed by a multidisciplinary team of medical mycologists and bioinformaticians to assess the potential presence of rare or emerging fungal pathogens not represented in current databases.
2. tNGS panels cover a wide range of known pathogens and are more cost-effective than mNGS; however, they may miss rare or emerging fungal infections.
3. In medical centers lacking diagnostic capabilities, perform basic microscopy and culture and refer specimens where possible. If urgent deterioration occurs, empirical management may proceed pending confirmation.
4. When mNGS/tNGS are used, interpret results in light of controls, specimen type, and colonization risk. Platforms, pipelines, databases, and cut-offs should be reported transparently to ensure reproducibility.

## Supplementary Information

Supplementary materials are only available at the official site of the journal (www.intern-med.com).

## Supplementary Material

Supplementary Material Details
