# Supplementary material for: Asian consensus on diagnostic algorithm for invasive pulmonary fungal diseases in high-burden settings
Source: J Transl Int Med. 2026 Aug 26;14(4):493–505. doi: 10.1515/jtim-2026-0065 (PMC13508831; doi:10.1515/jtim-2026-0065)
Supplement: Supplementary file 1 [file jtim-2026-0065_sm.zip › 1 JTIM-D-26-00276 Supplementary Material 1.pdf]

Supplementary Material 1

(Search strategy of literatures regarding IPFD)

(invasive pulmonary fungal disease OR invasive pulmonary fungal infection OR pulmonary fungal infection OR invasive pulmonary aspergillosis OR pulmonary aspergillosis OR pulmonary mucormycosis OR pulmonary cryptococcosis OR cryptococcal pneumonia OR pneumocystis pneumonia)
AND
(*Aspergillus* OR *Cryptococcus* OR *Pneumocystis* OR mucormycosis OR Mucorales OR *Rhizopus* OR *Mucor* OR *Lichtheimia* OR *Apophysomyces* OR *Cunninghamella* OR *Talaromyces* OR *Histoplasma* OR *Emergomyces*)
AND
(diagnos* OR mycological testing OR laboratory diagnosis)
AND
(guideline OR consensus OR recommendation* OR statement* OR review OR systematic review OR meta-analysis)
