# Supplementary material for: Asian consensus on diagnostic algorithm for invasive pulmonary fungal diseases in high-burden settings
Source: J Transl Int Med. 2026 Aug 26;14(4):493–505. doi: 10.1515/jtim-2026-0065 (PMC13508831; doi:10.1515/jtim-2026-0065)
Supplement: Supplementary file 1 [file jtim-2026-0065_sm.zip › 1 JTIM-D-26-00276 Supplementary Material 2.pdf]

Supplementary Material 2

Supplementary Table S1: Accessibility data of recommended IPFD diagnostic methods in high-burden Asian settings

Diagnostic category/subcategory	Overall (*n* = 191)		Lower-resource centers (< USD 3000) (*n* = 89)		Higher-resource centers (USD 3000–20,000) (*n* = 102)	
	n	**%**	***n***	**%**	***n***	**%**
Microscopy						
Calcofluor white	77	40.31%	39	43.82%	38	37.25%
Giemsa stain	137	71.73%	64	71.91%	73	71.57%
China/India ink	166	86.91%	80	89.89%	86	84.31%
Potassium hydroxide	162	84.82%	82	92.13%	80	78.43%
Silver stain	89	46.60%	43	48.31%	46	45.10%
Culture and fungal identification						
Overall	187	97.91%	88	98.88%	99	97.06%
Blood cultures when fungemia suspected	133	69.63%	65	73.03%	68	66.67%
Fungal culture methods						
Agar Niger	52	27.23%	20	22.47%	32	31.37%
Chromogen	50	26.18%	16	17.98%	34	33.33%
Laurential agar	9	4.71%	2	2.25%	7	6.86%
Potato dextrose agar	105	54.97%	46	51.69%	59	57.84%
SDA	161	84.29%	77	86.52%	84	82.35%
SDA + Chloramphenicol	136	71.20%	68	76.40%	68	66.67%
SDA + Gentamicin	81	42.41%	45	50.56%	36	35.29%
Selective agar (Chloramphenicol + Cycloheximide)	94	49.21%	46	51.69%	48	47.06%
Others	59	30.89%	26	29.21%	33	32.35%

Available tests for specific identification	166	86.91%	76	85.39%	90	88.24%
Automated identification (*i.e.*, VITEK, other commercial tests)	119	62.30%	55	61.80%	64	62.75%
Biochemical tests (classic mycology)	124	64.92%	54	60.67%	70	68.63%
DNA sequencing	60	31.41%	12	13.48%	48	47.06%
MALDI – TOF – MS	65	34.03%	21	23.60%	44	43.14%
Mounting medium	75	39.27%	38	42.70%	37	36.27%
Antifungal susceptibility tests?	154	80.63%	70	78.65%	84	82.35%
For yeasts	76	39.79%	46	51.69%	30	29.41%
For moulds	2	1.05%	1	1.12%	1	0.98%
For both	69	36.13%	21	23.60%	48	47.06%
Available antifungal susceptibility test technologies						
Broth microdilution, using CLSI standards	89	46.60%	32	35.96%	57	55.88%
Broth microdilution, using EUCAST standards	30	15.71%	9	10.11%	21	20.59%
E-test	71	37.17%	38	42.70%	33	32.35%
VITEK	94	49.21%	50	56.18%	44	43.14%
Serology	110	57.59%	44	49.44%	66	64.71%
Aspergillus spp.	102	53.40%	40	44.94%	62	60.78%
Candida spp.	73	38.22%	25	28.09%	48	47.06%
Histoplasma spp.	43	22.51%	25	28.09%	18	17.65%
Antigen detection	145	75.92%	66	74.16%	79	77.45%
Aspergillus overall	126	65.97%	58	65.17%	68	66.67%

Aspergillus LFD	46	24.08%	20	22.47%	26	25.49%
Aspergillus GM ELISA	112	58.64%	48	53.93%	64	62.75%
Aspergillus GM LFA	60	31.41%	28	31.46%	32	31.37%
Candida antigen	52	27.23%	21	23.60%	31	30.39%
Cryptococcus overall	124	64.92%	56	62.92%	68	66.67%
Cryptococcus LFA	97	50.79%	46	51.69%	51	50.00%
Cryptococcus LAT	89	46.60%	36	40.45%	53	51.96%
Histoplasma	39	20.42%	22	24.72%	17	16.67%
Beta-glucan	76	39.79%	36	40.45%	40	39.22%
**Molecular tests**	117	61.26%	46	51.69%	71	69.61%
Aspergillus PCR	79	41.36%	27	30.34%	52	50.98%
Candida PCR	86	45.03%	27	30.34%	59	57.84%
Pneumocystis PCR	71	37.17%	31	34.83%	40	39.22%
Mucorales PCR	56	0.293193717	22	0.247191011	34	0.33333333

Data are reported per participating centers/laboratories. Resource level categories were defined according to national per-capita GDP, as indicated in the column headings. Percentages are calculated within each column. Availability reflects access to the test rather than routine clinical use. IPFD: Invasive Pulmonary Fungal Diseases; SDA: Sabouraud Dextrose Agar; MALDI – TOF – MS: Matrix-Assisted Laser Desorption/Ionization Time-of-Flight Mass Spectrometry; CLSI: Clinical and Laboratory Standards Institute; EUCAST: European Committee on Antimicrobial Susceptibility Testing); LFD: Lateral-Flow Device; GM ELISA: Galactomannan Enzyme-Linked Immunosorbent Assay; GM LFA: Galactomannan Lateral Flow Assay; LAT: Latex Agglutination Test; PCR: Polymerase Chain Reaction; GDP: Gross Domestic Product.
